# *Riemerella anatipestifer* M949_1360 Gene Functions on the Lipopolysaccharide Biosynthesis and Bacterial Virulence

**DOI:** 10.1371/journal.pone.0160708

**Published:** 2016-08-08

**Authors:** Guijing Yu, Xiaolan Wang, Yafeng Dou, Shaohui Wang, Mingxing Tian, Jingjing Qi, Tao Li, Chan Ding, Yantao Wu, Shengqing Yu

**Affiliations:** 1 Shanghai Veterinary Research Institute, Chinese Academy of Agricultural Sciences, Shanghai 200241, China; 2 Yangzhou University, Yangzhou, People’s Republic of China; Centre National de la Recherche Scientifique, Aix-Marseille Université, FRANCE

## Abstract

*Riemerella anatipestifer* causes septicemic and exudative diseases in poultry, resulting in major economic losses to the duck industry. Lipopolysaccharide (LPS), as an important virulence factor in Gram-negative bacteria, can be recognized by the immune system and plays a crucial role in many interactions between bacteria and animal hosts. In this study, we screened out one LPS defective mutant strain RAΔ604 from a random transposon mutant library of *R*. *anatipestifer* serotype 1 strain CH3, which did not react with the anti-CH3 LPS monoclonal antibody 1C1 in an indirect enzyme-linked immunosorbent assay. Southern blot analysis confirmed that the genome of RAΔ604 contained a single Tn*4351* insert. Then, we found that the M949_1360 gene was inactivated by insertion of the transposon. Using silver staining and western blot analyses, we found that the LPS pattern of RAΔ604 was defective, as compared with that of the wild-type (WT) strain CH3. The mutant strain RAΔ604 showed no significant influence on bacterial growth, while bacterial counting and Live/dead BacLight Bacterial Viability staining revealed that bacterial viability was decreased, as compared with the WT strain CH3. In addition, the abilities of the mutant strain RAΔ604 to adhere and invade Vero cells were significantly decreased. Animal studies revealed that the virulence of the mutant strain RAΔ604 was decreased by more than 200-fold in a duck infection model, as compared with the WT strain CH3. Furthermore, immunization with live bacteria of the mutant strain RAΔ604 protected 87.5% ducks from challenge with *R*. *anatipestifer* serotype 1 strain WJ4, indicating that the mutant strain RAΔ604 could be used as a potential vaccine candidate in the future.

## Introduction

*Riemerella anatipestifer* is a Gram-negative, non-motile, non-spore-forming, rod-shaped bacterium, which belongs to *Flavobacteriaceae* rRNA superfamily V [1]. *R*. *anatipestifer* infection is primarily associated with acute septicemia and exudative inflammation in domestic and wild birds, causes the greatest mortality among duck diseases and has become ever more important in the poultry industry [2–3], however, different from proteobacteria, little is known about lipopolysaccharide (LPS) synthesis and virulence of this pathogen. To date, a total of 21 *R*. *anatipestifer* serotypes have been identified, but there is no significant cross-protection among them [4, 5]. In china, *R*. *anatipestifer* serotypes 1, 2, and 10 are responsible for most of the major outbreaks [6]. Besides, several virulence factors, including VapD [7], CAMP cohemolysin [8], and outer membrane protein A [9] have been identified.

LPS is a major component of the outer membrane of most Gram-negative bacteria and a major virulence factor that plays important roles in the integrity of the outer-membrane permeability and participates extensively in host-pathogen interplay [10–12]. LPS is composed of lipid A, a core polysaccharide (core-PS), and O-antigen repeats [13]. Lipid A represents the hydrophobic component of LPS which locates in the outer leaflet of the outer membrane, while core polysaccharides and O-antigen repeats are displayed on the surface of the bacterial cells [10, 14]. Lipid A, which is highly conversed in all the Gram-negative bacteria, is responsible for the major bioactivity of endotoxins [15]. The core-PS is known to have a role in maintaining outer membrane stability [16], while the O-antigen repeats provides the major antigenic variability of the cell surface [17]. The genes involved in the synthesis of LPS have been characterized in several species of bacteria [18]. For example, the *lpxD-fabZ-lpxA-lpxB* gene cluster required for lipid A biosynthesis has been identified in *Escherichia coli* and other bacteria [19, 20]. In *R*. *anatipestifer*, however, there were only three genes (AS87_04050, M949_1556, and M949_1603) associated with LPS synthesis characterized in our previous study [21–23]. In this study, a random transposon mutant library of *R*. *anatipestifer* CH3 strain [24] was screened for mutants that displayed reduced reactivity to the monoclonal antibody (MAb) 1C1, which was raised against CH3 LPS (anti-CH3 LPS MAb). This led to the identification of a novel M949_1360 gene associated with *R*. *anatipestifer* LPS biosynthesis. The roles of the M949_1360 gene in LPS biosynthesis and bacterial virulence were also characterized.

## Materials and Methods

### Ethics statement

One-day-old Cherry Valley ducks were purchased from Zhuanghang duck farm (Shanghai, China) and housed in cages under a 12-h light/dark cycle kept at a controlled temperature (28 to 30°C) with free access to food and water. This study was performed in strict accordance with the guidelines described in the Guide for the Care and Use of Laboratory Animals of the Institutional Animal Care and Use Committee of Shanghai Veterinary Research Institute, the Chinese Academy of Agricultural Sciences (CAAS). The protocol was approved by the Committee on the Ethics of Animal Experiments of Shanghai Veterinary Research Institute, CAAS (Permit Number: shvri-po-0242). To minimize the suffering, dying ducks were euthanized humanely with an intravenous injection of sodium pentobarbital at a dose of 120 mg/kg.

### Bacterial strains, plasmids and culture conditions

The bacterial strains and plasmids used in this study are listed in Table 1. *R*. *anatipestifer* wild-type (WT) strain CH3 (serotype 1) was isolated and preserved in our laboratory [25]. The mutant strain RAΔ604 was screened out from a random transposon mutant library that was previously constructed in our laboratory [24]. The *Escherichia coli–Flavobacterium johnsoniae* shuttle plasmid pCP29 and *E*. *coli* strain BW19851, which contains the plasmid pEP4351, were provided by Professor Mark J. McBride, University of Wisconsin-Milwaukee (Milwaukee, WI, USA). *R*. *anatipestifer* strains were cultured in tryptic soy broth (TSB, Difco, USA) or on tryptic soy agar (TSA, Difco, USA) at 37°C under an atmosphere of 5% CO_2_, and *E*. *coli* strains were grown at 37°C in Luria–Bertani (LB) broth or on LB plates. Antibiotics were used at the following concentrations when needed: ampicillin (100 μg/ml), chloramphenicol (30 μg/ml), erythromycin (0.5 μg/ml), kanamycin (50 μg/ml), and cefoxitin (5 μg/ml), gentamicin (100 μg/ml).

**Table 1:** Strains, plasmids, and primers used in this study.

Strains, plasmids or primers	Description	Source or references
**Strains**		
CH3	*Riemerella anatipestifer* wild-type strain (serotype 1)	[24]
RAΔ604	Mutant strain, derived from *Riemerella anatipestifer* CH3, in which the M949_1360 gene was inactivated by Tn4351 insertion.	This study
RAc604	Mutant RAΔ604 carrying plasmid pCP29-M949_1360	This study
*Escherichia coli* S17-1	lpir hsdR pro thi; chromosomal integrated RP4-2 Tc::Mu Km::Tn7	[24]
**Plasmids**		
pCP29	ColE1 ori; (pCP1 ori); Ap^r^(Em^r^); *E*. *coli-F*. *johnsoniae* shuttle plasmid	[24]
pCP29-M949_1360	Pcp29 containing *ompA* promoter and M949_1360 ORF, cfxA^r^ (Ap^r^)	This study
**Primers**		
RA 16S rRNA-F	5'-GAGCGGTAGAGTATCTTCGGATACT-3'	This study
RA 16S rRNA-R	5'-AATTCCTTTGAGTTTCAACCTTGCG-3'	This study
Tn4351-F	5'-TGGCACCTTTGTGGTTCTTAC-3'	[21]
Tn4351-R	5'-GAGAGACAATGTCCCCCTTTC-3'	[21]
RAΔ604com-F	5'-ACGCTCGAGATGAAGAAAATTCTTTTTATCGC-3' (*Xho*I site underlined)	This study
RAΔ604com-R	5'-ATTGCATGCTTACTTAATTATATTATGAATCC-3' (*Sph*I site underlined)	This study
RA DnaB P1	5'-AAACTCAGGCAAAGGTGGCAC-3'	[26]
RA DnaB P2	5'-TGTATGGTAGTTTTGATGCTTTCAA-3'	[26]
*E*. *coli* phoA P1	5'-CGATTCTGGAAATGGCAAAAG-3'	[26]
*E*. *coli* phoA P2	5'-CGTGATCAGCGGTGACTATGAC-3'	[26]
RA *ldh*-F	5'-AACTTCCGCTTGGTATGCAC-3'	[21]
RA *ldh*-R	5'-TAGCCGCAGTAGCGAATTTT-3'	[21]
M949_1359-F	5'-CAGTTTCGAAAAACGGGAAA-3'	This study
M949_1359-R	5'-CAATCCGATTCCACAACAAA-3'	This study
M949_1360-F	5'-ACCAGGCAGGATTGTCTTTC-3'	This study
M949_1360-R	5'-TGAATCCAATGCTCCAATGA-3'	This study
M949_1361-F	5'-TTTTGGTCTTCCACCCGTAG-3'	This study
M949_1361-R	5'-AAATCTCTGATGGGCACCTG-3'	This study

### Development of MAb 1C1 against *R*. *anatipestifer* LPS

MAb 1C1 against *R*. *anatipestifer* LPS was prepared in our laboratory previously [22]. Briefly, BALB/_C_ mice were subcutaneously immunized three times with 0.4% formalin inactivated *R*. *anatipestifer* CH3 at a dose of 10^8^ colony forming units (CFU), and the hybridoma technique was performed for the MAb development. Hybridomas were screened using indirect enzyme-linked immunosorbent assay (ELISA), in which plates were coated with purified CH3 LPS (10 ng/well). Positive hybridoma clones were identified and sub-cloned three times by limiting dilution. The hybridoma cells producing anti-CH3 LPS MAb 1C1 were obtained and selected for MAb production.

### Indirect ELISA

A random transposon mutant library of *R*. *anatipestifer* CH3 was constructed in our laboratory with *E*. *coli* strain BW19851 as the donor and *R*. *anatipestifer* CH3 as the recipient [24]. Anti-CH3 LPS MAb 1C1 was used to screen the library for mutant strains that showed no reactivity with the indirect ELISA. Briefly, 96-well ELISA plates were coated with whole cells of mutant at 10^9^ CFU/well in 50 μl carbonate-buffered saline (pH = 9.6). *R*. *anatipestifer* WT strain CH3 was used as a positive coating control. The plates were then dried overnight in a drying oven at 55°C. After three washes with phosphate-buffered saline (PBS, pH = 7.4), the plates were blocked with PBS-5% skim milk and incubated with MAb 1C1 as a primary antibody and horseradish peroxidase-conjugated anti-mouse IgG [Tiangen Biotech (Beijing) Co., Ltd., Beijing, China] as a secondary antibody. After the addition of 3,3',5,5'-tetramethylbenzidine [Tiangen Biotech (Beijing) Co., Ltd.] and stop buffer (2 M H_2_SO_4_), absorbance at an optical density (OD) of 450 nm (OD_450_) was obtained using a microplate spectrophotometer (Thermo Fisher Scientific, Inc., Waltham, MA, USA). Compared with the negative control (N), the mutant (S) showing an OD_450_ value of less than 2.1 (S/N< 2.1) was selected for further identification.

### Identification of the mutant strain RAΔ604

Southern blot analysis was performed to determine the single insertion of transposon Tn*4351* in the mutant strain. Briefly, genomic DNA of the mutant strain was extracted using the TIANamp Bacteria DNA kit [Tiangen Biotech (Beijing) Co., Ltd.] and digested with the restriction enzyme *Xba*I at 37°C for 2 h. After separated by gel electrophoresis (80 V, 80 min), the samples were transferred to a nitrocellulose (NC) membrane as described elsewhere [14]. Plasmid pEP4351 and genomic DNA of *R*. *anatipestifer* WT strain CH3 were used as positive and negative controls, respectively. Probes were prepared using the DIG DNA labeling and detection kit (Roche Diagnostics, Indianapolis, IN, USA), according to the manufacturer’s instructions.

The site of transposon insertion in the mutant strain was identified by inverse polymerase chain reaction (PCR) [27]. In brief, genomic DNA of the mutant strain was digested with restriction enzyme *Hind*III at 37°C for 3 h. After purification, the DNA fragment was ligated using T4 DNA Ligase [TaKaRa Biotechnology (Dalian) Co., Ltd., Dalian, China]. The Tn*4351*-specific primers TN-1 and IS4351 were used for PCR amplification. The resulting product was subjected to sequence analysis. The obtained DNA sequences were analyzed using the basic local alignment search tool (BLAST) algorithm (http://blast.ncbi.nlm.nih.gov/Blast.cgi) [28] to identify the site of transposon insertion.

Quantitative Real-time PCR (qPCR) was used to determine the transcriptional level of the flanking genes and target gene. Gene-specific primers were designed using Primer3 online software v.0.4.0 (http://bioinfo.ut.ee/primer3-0.4.0/) [29]. The expression levels of the upstream gene M949_1359, the inactivated gene M949_1360, and downstream gene M949_1361 were measured using respective primer pairs. The expression of the L-lactate dehydrogenase encoding gene (*ldh*) was measured as an internal control using the primer pair RA *ldh* F/RA *ldh* R [21]. All primers used in this study were listed in Table 1. Reactions were performed in triplicate and run on the Mastercycler ep realplex 4 apparatus (Eppendorf, Hamburg, Germany).

### Complementation of the mutant strain

To construct RAΔ604 complementation strain, the M949_1360 gene was amplified from *R*. *anatipestifer* CH3 using the primer pair RAΔ604com-F/RAΔ604com-R (Table 1). The PCR fragment and the plasmid pCP29 [30] were separately digested with *Xho*I and *Sph*I. Then, the final products were ligated and named pCP29-M949_1360. The expression of the M949_1360 gene was under the control of the *ompA* promoter. The plasmid pCP29-M949_1360 was transformed into competent *E*. *coli* S17-1 cells and the recombinant plasmid was introduced into cells of the mutant strain RAΔ604 by conjugation and transduction. The transconjugants were selected using TSA containing cefoxitin and kanamycin, and identified by PCR amplification using primers RAΔ604com-F/RAΔ604com-R, RA 16S rRNA-F/RA 16S rRNA-F, RA DnaB P1/RA DnaB P2 [26] and *E*. *coli* phoA P1/ *E*. *coli* phoA P2 [26] (negative control). The positive strain was named cRA604.

### Bacterial growth curves and viability assessment

*R*. *anatipestifer* CH3, RAΔ604, and cRA604 were cultured in TSB at 37°C with shaking at 200 rpm until the mid-logarithmic phase. At a ratio of 1:100, the bacterial cultures were diluted respectively into fresh TSB and grown at 37°C with shaking at 200 rpm [31]. Bacterial growth was measured by monitoring at OD_600_ at 1-h intervals using a spectrophotometer (Bio-Rad Laboratories, Inc., Hercules, CA, USA). A probability (*p*) value of <0.05 was considered statistically significant. This assay was performed in triplicate.

For assessment of bacterial viability, bacterial counting and Live/dead BacLight Bacterial Viability staining were performed. Briefly, each strain was adjusted to an OD_600_ value of 0.1 using TSB. Then, each 4 ml of the bacterial suspension were added to the wells (for bacterial counting), or wells containing a glass coverslip in six-well plates (for Live/dead BacLight Bacterial Viability staining) and incubated at 37°C under an atmosphere of 5% CO_2_ for 24 h. Bacteria were respectively counted, or stained with Live/dead BacLight Bacterial Viability staining reagent (Invitrogen Corporation, Carlsbad, CA, USA) according to the manufacture’s protocol at 24 h incubation. For the bacterial counting, each strain was provided with three replicates, diluted to appropriate concentrations and plated on TSA, then incubated at 37°C under an atmosphere of 5% CO_2_ for 24 h for calculating bacterial CFU. The image profiles of the bacterial shapes were observed by fluorescence microscopy as described previously [24].

### Serum bactericidal assay

Complement-sufficient duck sera without anti-*R*. *anatipestifer* antibody were from naive ducks, which were pooled from equal volumes of sera collected from 10 16-day-old healthy Cherry Valley ducks. The sera were filter-sterilized (0.22 μm) for the bactericidal assay. Briefly, pooled sera were diluted to 50% in PBS. Each bacterial strain was adjusted to 10^6^ CFU in 10 μl of PBS, then added into 190 μl of 50% diluted pooled sera, pooled sera without dilution, or heat-inactivated pooled sera (negative control) and incubated for 30 min at 37°C. The reaction products were serial diluted (10-fold), plated on TSA, and counted after incubation for 24 h at 37°C [21]. The bactericidal rates were calculated as: (1-bacterial CFU in pooled sera/bacterial CFU in the inactivated sera) × 100%.

### LPS extraction and silver staining

LPS was isolated from *R*. *anatipestifer* WT strain CH3, the mutant strain RAΔ604, and the complementation strain cRAΔ604, respectively, using LPS Extraction Kit [iNtRONbio (Korea) Co., Ltd.] according to the manufacture’s protocol. Briefly, bacterial cells in 2 ml culture were harvested by centrifugation at 1,000 ×g for 5 min. The pellets were then suspended in 1 ml Lysis Buffer, added with 200 μl chloroform, and placed at 15 to 30°C for 5 min. The samples were centrifugated at 2,000 ×g for 10 min at 4°C. The LPS in the aqueous phase was collected and added 800 μl Purification Buffer. After centrifugated at 1,000 ×g for 10 min at 4°C and washed twice with 70% ethanol, the LPS pellets were dissolved with 50 μl ddH_2_O [32]. Purified LPS was re-suspended in 2× loading buffer [50 mM of Tris-HCl (pH 6.8), containing 2% sodium dodecyl sulfate (SDS), 4% 2-mercaptoethanol, 10% glycerol, and 0.004% bromophenol blue] and boiled at 100°C for 10 min. Then, 10 μl of the sample was added to each well and subjected to sodium dodecyl sulfate polyacrylamide gel electrophoresis (SDS-PAGE) on a Mini Protein II gel system (Bio-Rad Laboratories, Inc.) with 15% polyacrylamide in the separating gel. After electrophoresis (100 V, 2 h), the LPS on the gel was subjected to silver staining [21].

### Western blot

Western blot was performed to verify the binding activity of anti-CH3 LPS MAb 1C1 with *R*. *anatipestifer* WT strain CH3, the mutant strain RAΔ604, and the complementation strain cRAΔ604. For each strain, 1 ml of freshly cultured bacteria was harvested by centrifugation (5000 ×g, for 5 min). The collected bacterial cells were re-suspended in 100 μl of PBS, boiled at 100°C for 10 min, and then separated by SDS-PAGE. After transfer to a NC membrane (Millipore Corporation, Billerica, MA, USA), western blot analysis was performed using anti-CH3 LPS MAb 1C1 (1:1000 dilution) as the primary antibody and rabbit anti-mouse IgG (LI-COR Biosciences, Lincoln, NE, USA) as the secondary antibody. Binding was determined using the Odyssey infrared imaging system (LI-COR Biosciences).

### Determination of bacterial virulence

The bacterial median lethal dose (LD_50_) was measured to evaluate virulence. Fifty 16-day-old Cherry Valley ducks were randomly divided into five groups (10 ducks per group) for each strain and inoculated intramuscularly with bacteria at 10^6^, 10^7^, 10^8^, 10^9^, or 10^10^ CFU, respectively [22]. For each group, the number of deaths was recorded for 7 days post-infection (DPI). Bacterial LD_50_ values were calculated using the modified Karber’s method [33].

To determine whether inactivation of the M949_1360 gene was associated with bacterial virulence and had an influence on the bacterial survival *in vivo*, 16-day-old Cherry Valley ducks were randomly separated to three groups (six ducks per group) and injected intramuscularly with *R*. *anatipestifer* WT strain CH3, the mutant strain RAΔ604, or the complementation strain cRAΔ604, respectively, at 10^9^ CFU in 0.5 ml PBS. Blood samples were collected daily for 5 days, diluted to appropriate concentrations, plated in triplicate on TSA, and then incubated at 37°C under an atmosphere of 5% CO_2_ for 24 h for bacterial counting.

### Adherence and invasion assays

Bacterial adherence and invasion were investigated using Vero cells (ATCC CCL-81; ATCC, Manassas, VA, USA) as described previously [9, 34]. Confluent monolayers of Vero cells were prepared in wells of 24-well plates in Dulbecco’s modified Eagle’s medium (DMEM; Biowest, Nuaillé, France) containing 10% fetal bovine serum. *R*. *anatipestifer* WT strain CH3, the mutant strain RAΔ604, and the complementation strain cRAΔ604 were inoculated respectively into separate wells at 50 multiplicity of infection and incubated at 37°C under an atmosphere of 5% CO_2_ for 1.5 h. Then, the cells were thoroughly washed with PBS. After trypsin digestion, the cell suspensions were 10-fold serially diluted and plated on TSA for bacterial counting. For the invasion assay, after washing the cells, 1 ml of DMEM containing 100 μg/ml gentamicin was added to each well and the plate was incubated for 1 h. With the same process of the adherence assay, samples were collected, diluted, plated in triplicate on TSA, and incubated at 37°C under an atmosphere of 5% CO_2_ for 24 h.

### Animal immunization experiment

To evaluate the potential use of the attenuated mutant strain RAΔ604 as a live vaccine candidate, Cherry Valley ducks were immunized twice at day 7 and 14 respectively with alive RAΔ604 at a dose of 5×10^8^ CFU/ml in 0.5 ml PBS [35]. At day 14 post second immunization, the immunized ducks were challenged with *R*. *anatipestifer* strain WJ4 (serotype 1) at 10 LD_50_. Non-immunized ducks were injected with WJ4 as controls. Ducks were monitored daily for clinical symptoms and death until 7 DPI.

### Statistical analyses

Statistical analyses were performed using the GraphPad Software (GraphPad Software Inc., La Jolla, CA, USA). One-way analysis of variance (ANOVA) was used for analyses of growth curves, bacterial loads in blood, serum bactericidal efficiency, adhesion capacity, and invasion data, and two-way ANOVA was performed for analysis of the qPCR results. The results are presented as mean values. A *p* value < 0.05 was considered statistically significant.

## Results

### Characterization of the mutant strain RAΔ604 and the complementation strain cRAΔ604

**Fig 1:**
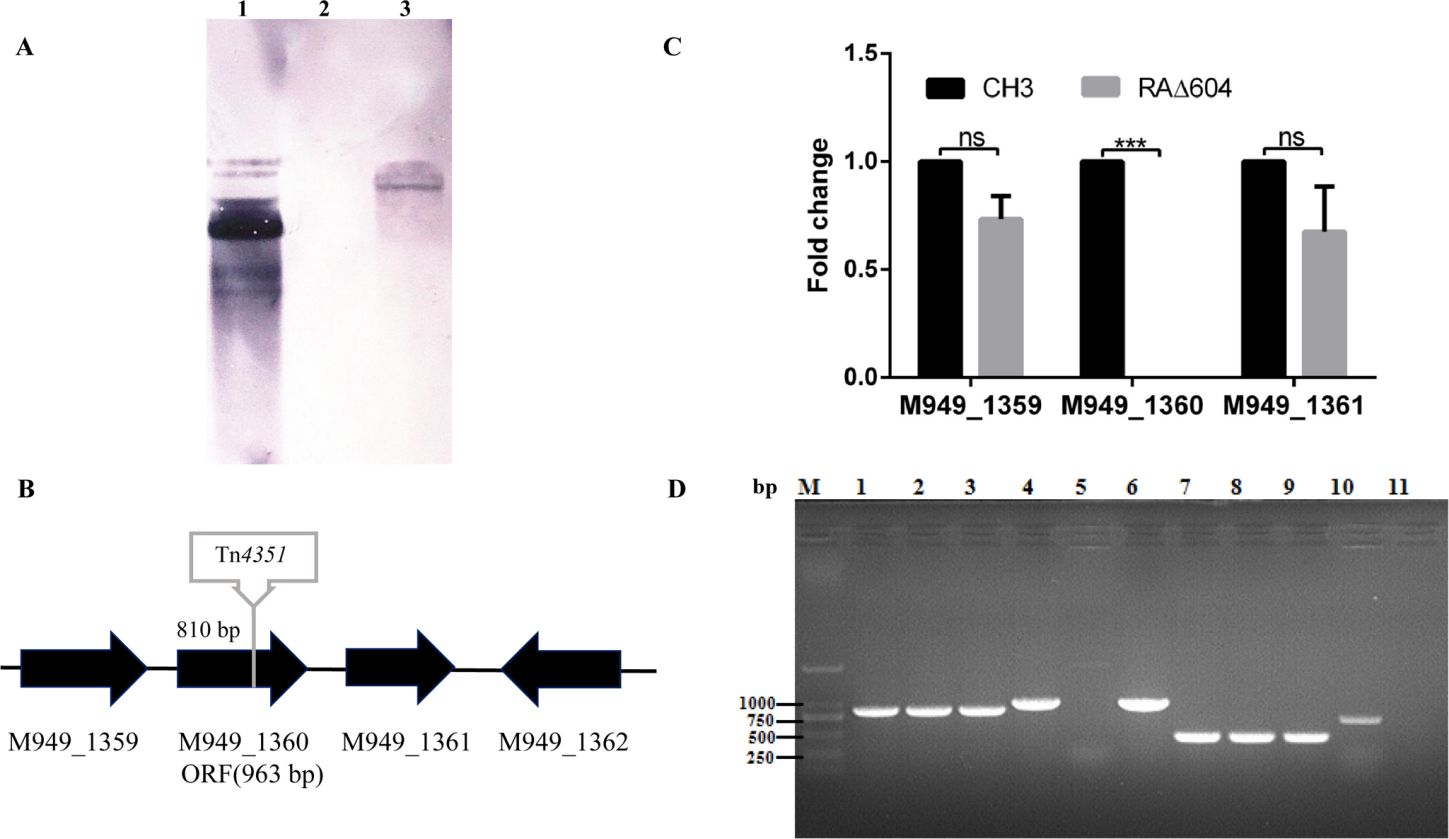
**Characterization of *R*. *anatipestifer* mutant strain RAΔ604 and complementation strain cRAΔ604** (A) Southern blot analysis. Lane 1: PEP*4351* (positive control); Lane 2: *R*. *anatipestifer* CH3 (negative control); Lane 3: mutant strain RAΔ604. (B) Schematic chart of Tn*4351* insertion in RAΔ604 chromosome at 810 bp of the M949_1360 gene. The flanking genes M949_1359, M949_1361, and M949_1362 were annotated as hypothetical protein, glycosyl transferase group 1 and lipopolysaccharide-modifying protein respectively. (C) qPCR analysis. Changes in mRNA levels are expressed as fold expression and calculated using the comparative CT (2^-ΔΔCT^) method. The expression of M949_1360 was inactivated in RAΔ604. However, no significant difference was shown in expression levels of M949_1359 and M949_1361 between *R*. *anatipestifer* CH3 and RAΔ604 (ns, *p* > 0.05). Error bars represent standard deviations from three replicates (***, *p* < 0.01). (D) Identification of the complementation strain cRAΔ604 by PCR. Takara DL2000 marker; lanes 1–3: *R*. *anatipestifer* 16S rRNA was amplified from the WT strain CH3 (lane 1), the mutant strain RAΔ604 (lane 2), and the complementation strain cRAΔ604 (lane 3), showing a 744-bp fragment of 16S rRNA; lanes 4–6: 963-bp fragment of M949_1360 gene was amplified from the WT strain CH3 (lane 4), and the complementation strain cRAΔ604 (lane 6), no 963-bp fragment of M949_1360 gene was amplified from the mutant strain RAΔ604 (lane 5); lanes 7–11: a 459-bp fragment of *R*. *anatipestifer* dnaB gene, but not a *E*. *coli* phoA gene was amplified from the WT strain CH3 (lane 7), the mutant strain RAΔ604 (lane 8) and the complementation strain cRAΔ604 (lane 9); lane 10: a 720-bp fragment of *E*. *coli* phoA gene was amplified from *E*. *coli* S17-1 strain; lane 11: distilled water, as a negative control.

The mutant strain RAΔ604 was screened out from the random transposon mutant library, which showed defective reactivity with anti-CH3 LPS MAb 1C1 in an indirect ELISA. RAΔ604 was confirmed to have a single Tn*4351* insertion in the genome by Southern blot analysis (Fig 1A). Sequencing of the cloned DNA fragment revealed that the insertion site was located at position 1,396,467 in the complete genome sequence of CH3, or at 810 bp of the M949_1360 gene in CH3 strain (GenBank CP006649.1) [36]. The M949_1360 gene is 963 nucleotides in length, coding for 320 amino acids. BLAST analysis of the nucleotide sequence shows that the M949_1360 gene was highly conserved in *R*. *anatipestifer*, which exhibited 100% identity with RA-CH-1 (GenBank: AFR35494.1) and 98% identity with Yb2 (GenBank: AKQ40115.1), 153 (GenBank: AKP71264.1), 17 (GenBank: AKP69367.1), RA-CH-2 (GenBank: AGC39923.1), ATCC 11845 = DSM 15868 (GenBank: AFD56156.1), RA-GD (GenBank: ADZ12343.1), and DSM 15868 (GenBank: ADQ82156.1). The protein coding by M949_1360 exhibited sequence similarity to the LPS core biosynthesis protein RfaS of *Chryseobacterium koreense* (GenBank: WP_048499682.1, 46%), the LPS biosynthesis protein of *Capnocytophaga ochracea* (GenBank: WP_041546932.1, 44%) and *Capnocytophaga sp*. *oral taxon 380* (GenBank: WP_034542854.1, 43%). HHPRED search (http://toolkit.tuebingen.mpg.de/hhpred) found that a very high match of this protein with the crystal structure of PimA/B GDP-mannose-dependent alpha-(1–2)-phosphatidylinositol mannosytransferase in *Mycobacteria*. The PimA/B plays a role in biosynthesis of phosphatidylinositol mannosides (PIMs), which is an essential enzyme for the initial mannosylation of phosphatidylinositol [37]. PIMs are cell wall derived lipoglycans important for host pathogen interactions in *Mycobacteria* [38]. We speculated that the M949_1360 gene might effect on the biosynthesis of PIMs.

Expression levels of the upstream M949_1359 gene and downstream M949_1361 gene were determined by qPCR using the *ldh* as an internal control. With three biological repeats, the data analyses showed no significant changes between the *R*. *anatipestifer* WT strain CH3 and the mutant strain RAΔ604, indicating that the insertion of Tn*4351* in the M949_1360 gene presented no polar effect on the expression of the upstream and downstream genes. Expression of the Tn4351-disrupted M949_1360 gene was not detectable in the mutant strain RAΔ604 by qPCR (Fig 1C).

Plasmid pCP29-M949_1360, which carries an M949_1360 gene under the control of the *R*. *anatipestifer ompA* promoter, was constructed and used for complementation of the mutant strain RAΔ604. Complementation strain was identified by PCR amplification (Fig 1D) and designated as cRAΔ604.

### LPS pattern analysis

**Fig 2:**
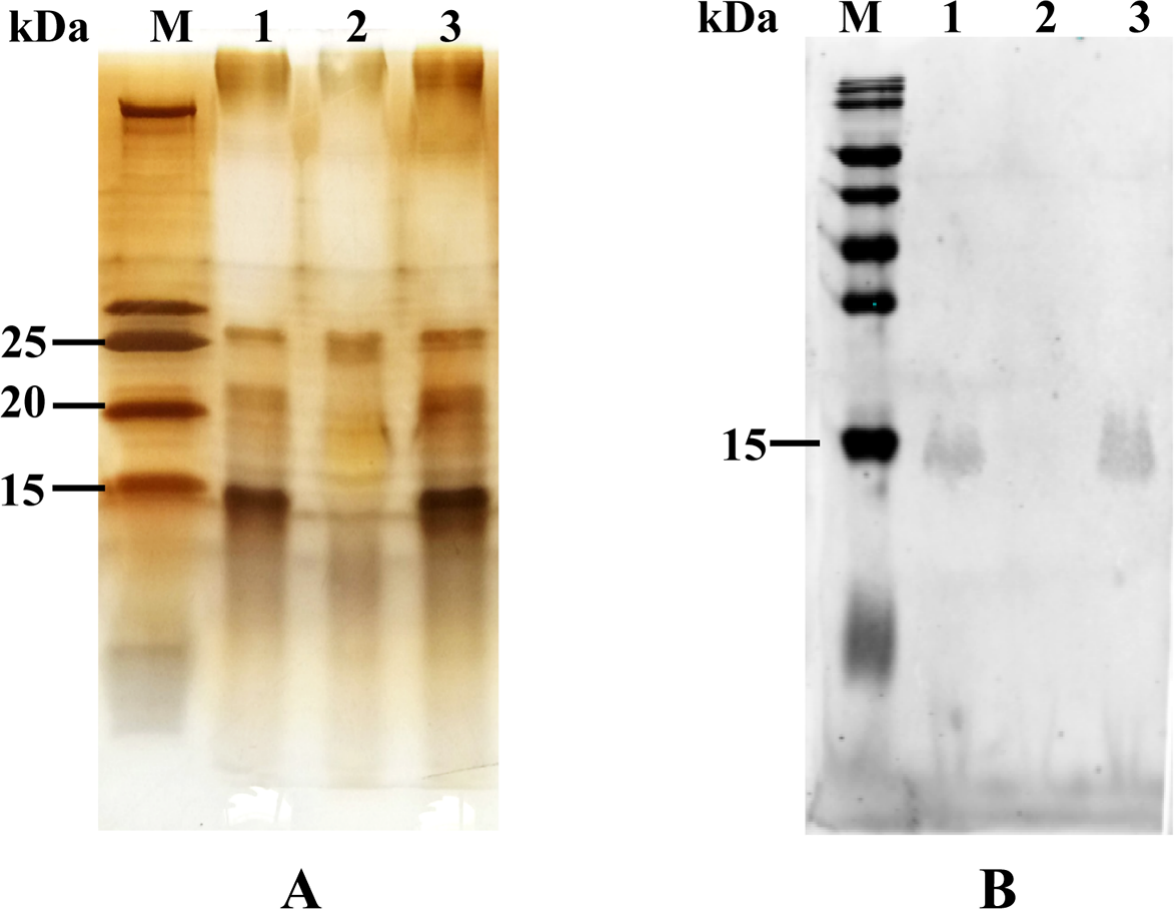
LPS structure analysis. (A) Silver staining of purified LPS. Each lane contained 0.5 μg of purified LPS. Lane M: Prestained Protein Ladder (Thermo Fisher Scientific, Inc.); Lane 1: *R*. *anatipestifer* CH3 LPS; Lane 2: The mutant strain RAΔ604 LPS; Lane 3: The complementation strain cRAΔ604 LPS. (B) Western blot analysis. Each lane contained 10^8^−10^9^ CFU of bacteria in 10 μl volume. After SDS-PAGE and transferred to a NC membrane, the NC membrane was incubated with anti-CH3 LPS MAb 1C1 (1:2000 dilution). Lane M: Prestained Protein Ladder (Thermo Fisher Scientific, Inc.); Lane 1: *R*. *anatipestifer* WT strain CH3; Lane 2: The mutant strain RAΔ604 LPS; Lane 3: The complementation strain cRAΔ604.

To examine whether the LPS pattern of RAΔ604 on the SDS-PAGE had changed, *R*. *anatipestifer* LPS was isolated from the WT strain CH3, the mutant strain RAΔ604, and the complementation strain cRAΔ604, separated by SDS-PAGE, and silver stained. As shown in Fig 2A, compared with the WT strain CH3, the mutant strain RAΔ604 displayed one band at 25 kDa, but the 20 kDa, and the 15 kDa bands were absent. Therefore, inactivation of the M949_1360 gene changed the phenotype of LPS, suggesting that this gene plays a role on LPS biosynthesis. The LPS phenotype was recovered in the complementation strain cRAΔ604.

### Western blot

Western blot analysis was used to determine the reactivity of *R*. *anatipestifer* strains with anti-CH3 LPS MAb 1C1. As shown in Fig 2B, the WT strain CH3 showed the binding band with the anti-LPS MAb, whereas the mutant strain RAΔ604 lost reactivity. The complementation strain cRAΔ604 recovered reactivity to anti-CH3 LPS MAb 1C1.

### Deletion of the M949_1360 gene decreased bacterial viability

**Fig 3:**
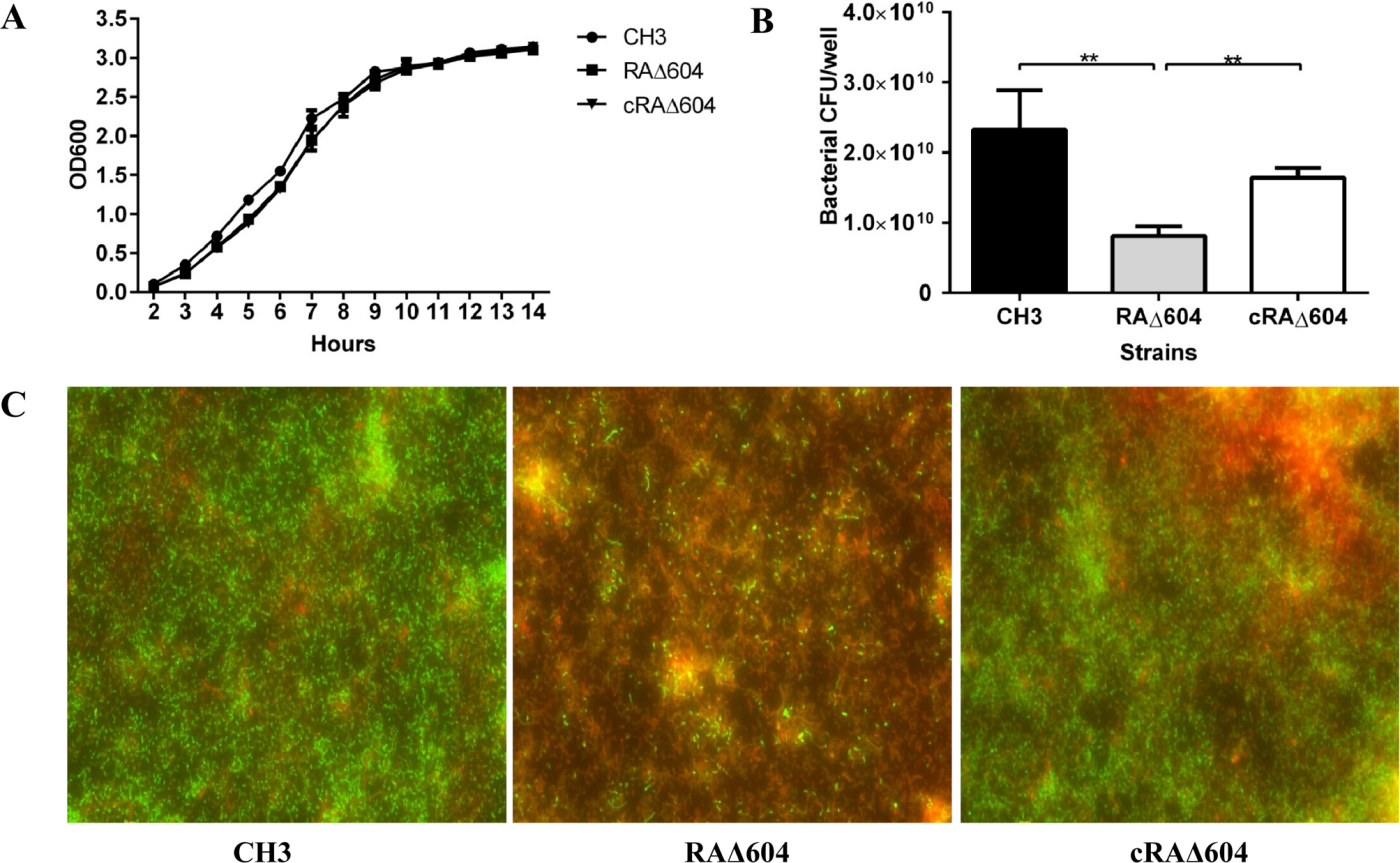
Bacterial growth curves and viability assessment. (A) There were no significant differences in bacterial growth curves of *R*. *anatipestifer* WT strain CH3, RAΔ604, and cRAΔ604 measured as OD_600_ values at 1-h intervals. The data are presented as the means of three repeats. Error bars represent standard deviations. (B) Bacterial viability was assessed by bacterial CFU counting at 24 h incubation. About 3-fold less CFU counting of the mutant strain RAΔ604, compared to the WT strain CH3. The complementation strain cRAΔ604 recovered the bacterial CFU counting. (C) Live/dead BacLight Bacterial Viability staining reagent was used to view live and dead bacteria. Live cells can be stained as green fluorescent and dead cells can be stained as red fluorescent. Using NIS-Elements Viewer software, we merged the photos of green fluorescent and red fluorescent together (400×).

As shown in Fig 3A, the mutant strain RAΔ604 showed no significant difference in bacterial growth during culture in TSB, as compared with the WT strain CH3 and the complementation strain cRAΔ604. However, the mutant strain RAΔ604 (counted as 8.12×10^9^ CFU/well) showed about 3-fold less CFU than the WT strain CH3 (counted as 2.9×10^10^ CFU/well) (Fig 3B). Moreover, Live/dead BacLight Bacterial Viability staining of bacteria after 24 h of incubation showed a significant increase in the abundance of dead cells of the mutant strain RAΔ604, as compared with the WT strain CH3 (Fig 3C). The complementation strain cRAΔ604 recovered survival ability *in vitro*. These results suggest that the M949_1360 gene may participate in bacterial viability.

### Bacterial sensitivity to normal duck sera

**Fig 4:**
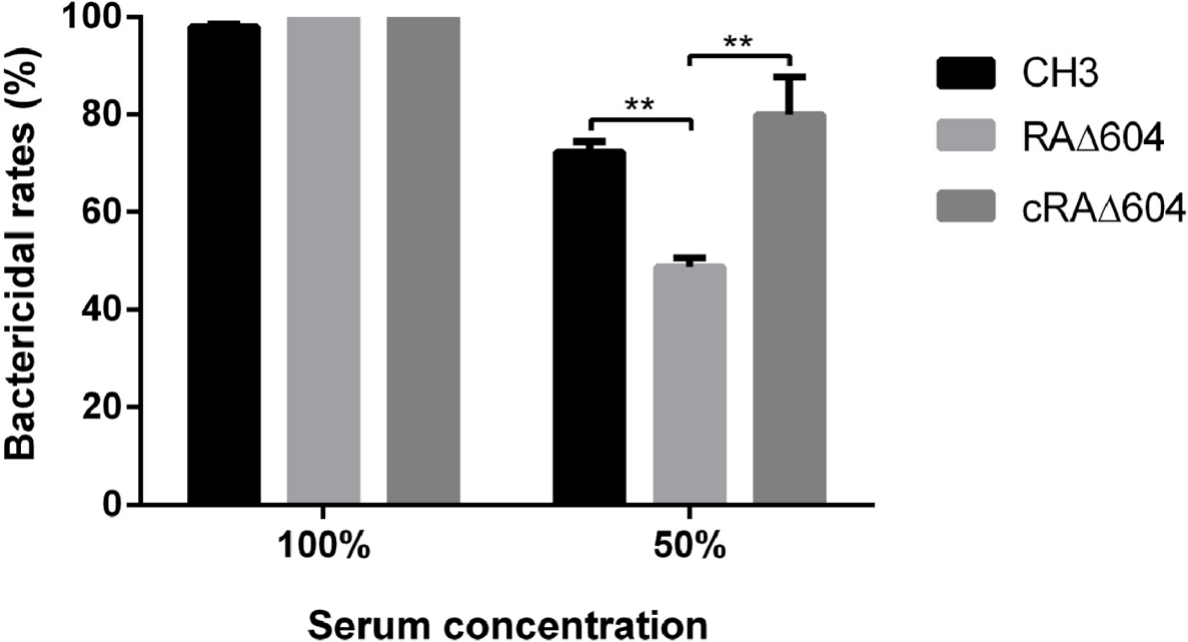
Serum bactericidal experiment. Bacteria were incubated with pooled normal duck sera at 50% dilution or without dilution at 37°C for 30 min. The heat-inactivated pooled duck sera were used as a control. The mutant strain RAΔ604 displayed significantly greater resistance to normal duck serum as compared to the WT and cRAΔ604 strains. The error bars represent mean ± standard deviations from three independent experiments (**, *p* < 0.05).

A bactericidal assay was performed to determine whether the M949_1360 gene is involved in bacterial resistance to normal serum. As shown in Fig 4, after incubation with 50% normal duck serum, the bactericidal rates for the WT strain CH3 and the mutant strain RAΔ604 were 72.3% and 48.7%, respectively, indicating that the mutant strain RAΔ604 displayed significantly greater resistance to normal duck serum than the WT strain CH3. Serum resistance was restored in the complementation strain cRAΔ604.

### Deletion of M949_1360 gene attenuated bacterial virulence

To determine whether the M949_1360 gene was associated with bacterial virulence, the bacterial LD_50_ was determined. Results showed that the LD_50_ for the WT strain CH3, the mutant strain RAΔ604, and the complementation strain cRAΔ604 were 4.46×10^7^ CFU, >10^10^ CFU, and >10^10^ CFU, respectively. Therefore, the virulence of RAΔ604 was attenuated more than 200-fold, as compared with the WT strain CH3. Complementation of M949_1360 gene did not restore the bacterial virulence of cRAΔ604 (Table 2).

**Table 2:** Determination of bacterial LD_50_ values.

Strains	LD_50_ (CFU)
CH3	4.46×10^7^
RAΔ604	>10^10^
cRA604	>10^10^

**Fig 5:**
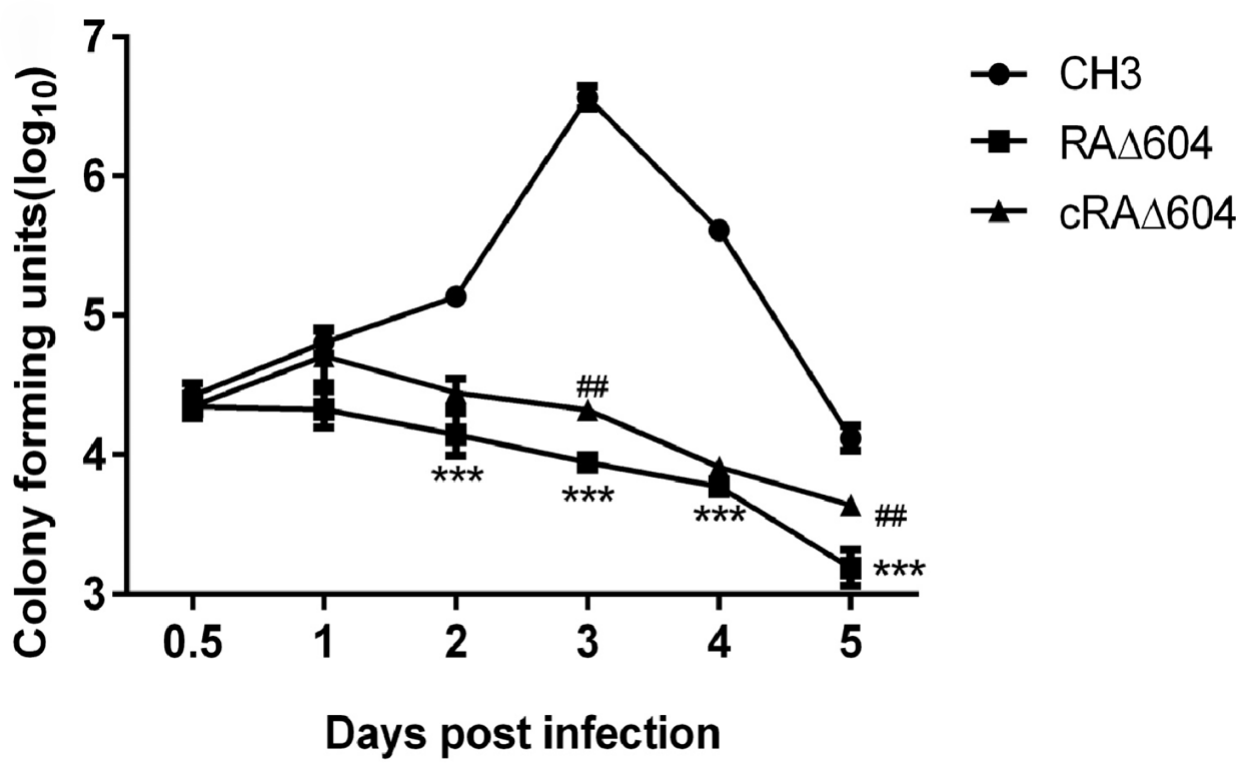
Bacterial loads in blood of infected ducks. Blood samples were collected at 0.5, 1, 2, 3, 4, and 5 DPI and bacterial concentrations were measured. Statistical difference between *R*. *anatipestifer* WT strain CH3 and the mutant strain RAΔ604 was marked by asterisks; The significance of difference between cRAΔ604 and RAΔ604 were marked by pound signs. Each point represents the mean ± standard deviation. (***, *p* < 0.01; ##, *p* < 0.05).

We further measured the bacterial loads in the blood of ducks infected with the WT strain CH3, the mutant strain RAΔ604, and the complementation strain cRAΔ604 daily for 5 DPI. As shown in Fig 5, the bacterial loads in the blood of ducks infected with the WT strain CH3 had increased with time and peaked at day 3 DPI. However, the bacterial loads in the blood of ducks infected with the mutant strain RAΔ604 gradually decreased with time by more than 10-fold at 5 DPI. Complementation of the M949_1360 gene restored part of the bacterial loads in the blood of ducks infected with cRAΔ604. These results suggest that the M949_1360 gene was associated with bacterial virulence.

### Inactivation of the M949_1360 gene decreased bacterial adherence and invasion capacities

**Fig 6:**
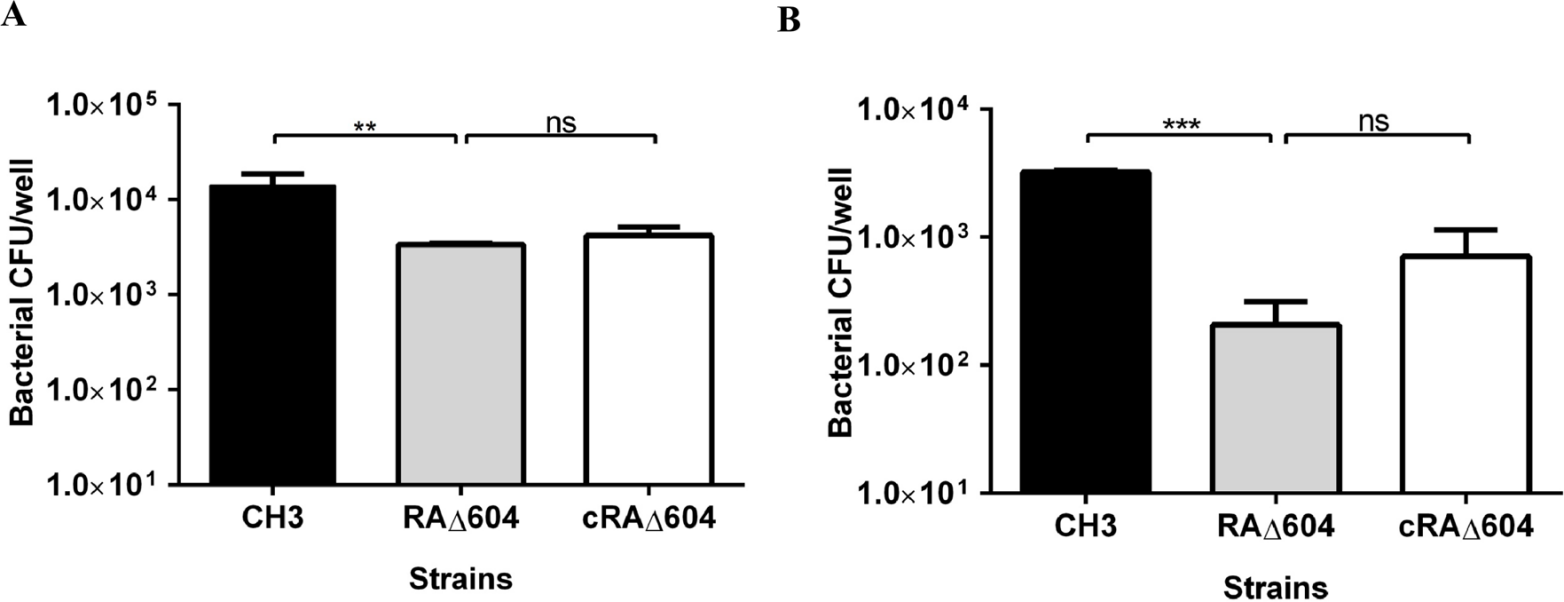
Bacterial adherence and invasion assays. (A) Adherence assay. (B) Invasion assay. Each point represents the mean ± standard deviation. Both adherence and invasion capacities of the mutant strain RAΔ604 were decreased, as compared with those of *R*. *anatipestifer* WT strain CH3 (***, *p* < 0.01; **, *p* < 0.05). The complementation strain cRAΔ604 partially recovered adherence and invasion capacities (ns, *p* > 0.05).

Vero cells were used to determine bacterial adherence and invasion abilities. The results showed that the bacterial adherence and invasion abilities of the mutant strain RAΔ604 were decreased, as compared with the WT strain CH3, indicating that the M949_1360 gene plays a role in bacterial invasion. The complementation strain cRAΔ604 partially recovered adherence and invasion capacities (Fig 6A and 6B).

### Animal vaccination experiment

Since the virulence of the mutant strain RAΔ604 was significantly attenuated, the potential use of RAΔ604 as a live vaccine candidate was evaluated. The results showed that RAΔ604 vaccinated ducks were 85.7% (7/8) protected from WJ4 challenge at 10 LD_50_, indicating that RAΔ604 induced protection against virulent bacterial challenge (Table 3).

**Table 3:** Animal immunization experiment.

Immunization	Challenge strains*	Protection rate
RAΔ604 vaccinated	WJ4	7/8
Non-vaccinated	WJ4	0/8

*The dose of challenge strains was 10 LD_50_.

## Discussion

Tn*4351* transposon insertion technology was successfully applied in our previous studies, which found that a total of 33 genes were involved in *R*. *anatipestifer* biofilm formation [24]. Moreover, three genes involved in *R*. *anatipestifer* LPS biosynthesis were identified by transponson mutagenesis [21–23]. In this study, the mutant strain RAΔ604 did not react with anti-CH3 LPS MAb 1C1, which was screened out using an indirect ELISA, and showed decreased bacterial viability, attenuated bacterial virulence, and increased resistance to normal duck serum, as compared with *R*. *anatipestifer* WT strain CH3.

The M949_1360 gene was determined to be inactivated in the mutant strain RAΔ604. A BLAST search showed that the M949_1360 gene shared 98%–100% identity with other *R*. *anatipestifer* strains in the GenBank database, indicating that the M949_1360 gene is highly conserved among *R*. *anatipestifer* strains. The M949_1360 gene encodes a protein that exhibits sequence similarity to the LPS core biosynthesis protein RfaS of *Chryseobacterium koreense*. The *rfaS* gene has been reported to be involved in production of a rough form of LPS core in *E*. *coli* K-12 [39, 40]. In this study, we found that the M949_1360 gene played a role in LPS synthesis of *R*. *anatipestifer*, based on the silver staining results of the defective LPS profile of RAΔ604, as compared to the WT strain CH3. Western blot analysis further revealed that RAΔ604 did not react with the anti-CH3 LPS MAb 1C1, indicating that the M949_1360 gene plays a role in LPS synthesis and antigenicity. With deletion of the M949_1360 gene, the defect LPS changed the stability of the outer membrane of the cells. Some studies revealed that LPS is crucial during growth and for antibiotic resistance [41, 42], which may explain why the mutant strain RAΔ604 reduced viability of *R*. *anatipestifer*.

Both *in vitro* and *in vivo* experiments were used to determine the function of the M949_1360 gene in bacterial pathogenicity. *In vivo*, according to the consequence of LD_50_, virulence of the mutant strain RAΔ604 was attenuated by more than 200-fold, as compared with *R*. *anatipestifer* CH3. Within 5 DPI of RAΔ604, the bacterial load in the blood of the infected ducks had significantly decreased with time, as compared with infection by *R*. *anatipestifer* CH3. These results imply that the M949_1360 gene is an important virulence gene in *R*. *anatipestifer*. *In vitro*, the ability of the mutant strain RAΔ604 to adhere to and invade Vero cells was compared with that of *R*. *anatipestifer* CH3. Adherence of bacteria to target cells of a susceptible host is considered the first step of successful colonization and has been associated with bacterial virulence [43, 44]. Also, invasion of bacterial pathogens has been shown to involve interactions between the bacterial surface and host cell receptors [45]. These results suggest that the M949_1360 gene may be a factor affecting the ability of *R*. *anatipestifer* to survive and proliferate in host cells.

The complement system is a critical part of host defense against microbial infection. Similarly, the O-antigenic polysaccharide moiety of LPS may participate in the combination of complement [46]; In our current study, the mutant strain RAΔ604 resisted activation of the complement pathway and displayed elevated levels of bacterial survival in duck serum, therefore, we speculated that the encoded protein of the M949_1360 gene contain a region that can bind to the complement combining site, thereby preventing activation of the complement system [47], however, the complement regulatory function of the M949_1360 gene should be further investigated. Moreover, animal experiments demonstrated that the attenuated mutant strain RAΔ604 had effectively protected the ducks from challenge with the same serotype 1 strain WJ4, suggesting that the mutant strain RAΔ604 was suitable as a vaccine candidate. In conclusion, the M949_1360 gene is involved in LPS biosynthesis and bacterial pathogenicity. This study provides a new basis for future prevention and control strategies against *R*. *anatipestifer* infection.
